# Establish immune-related gene prognostic index for esophageal cancer

**DOI:** 10.3389/fgene.2022.956915

**Published:** 2022-08-09

**Authors:** Caiyu Guo, Fanye Zeng, Hui Liu, Jianlin Wang, Xue Huang, Judong Luo

**Affiliations:** ^1^ Department of Radiotherapy, The Affiliated Changzhou No. 2 People’s Hospital of Nanjing Medical University, Changzhou, China; ^2^ Department of Radiotherapy, Graduate School of Dalian Medical University, Dalian, China; ^3^ Second Department of Medical Oncology, The Fourth Affiliated Hospital of Xinjiang Medical University, Urumqi, China; ^4^ School of Computer Science and Technology, Nanjing Tech University, Nanjing, China; ^5^ Department of Radiotherapy, Changzhou Tumor Hospital, Changzhou, China

**Keywords:** esophageal cancer, RNA-seq data, immune-related genes, prognostic model, overall survival, immune-related function

## Abstract

**Background:** Esophageal cancer is a tumor type with high invasiveness and low prognosis. As immunotherapy has been shown to improve the prognosis of esophageal cancer patients, we were interested in the establishment of an immune-associated gene prognostic index to effectively predict the prognosis of patients. Methods: To establish the immune-related gene prognostic index of esophageal cancer (EC), we screened 363 upregulated and 83 downregulated immune-related genes that were differentially expressed in EC compared to normal tissues. By multivariate Cox regression and weighted gene coexpression network analysis (WGCNA), we built a prognostic model based on eight immune-related genes (IRGs). We confirmed the prognostic model in both TCGA and GEO cohorts and found that the low-risk group had better overall survival than the high-risk group. Results: In this study, we identified 363 upregulated IRGs and 83 downregulated IRGs. Next, we found a prognostic model that was constructed with eight IRGs (OSM, CEACAM8, HSPA6, HSP90AB1, PCSK2, PLXNA1, TRIB2, and HMGB3) by multivariate Cox regression analysis and WGCNA. According to the Kaplan–Meier survival analysis results, the model we constructed can predict the prognosis of patients with esophageal cancer. This result can be verified by the Gene Expression Omnibus (GEO). Patients were divided into two groups with different outcomes. IRGPI-low patients had better overall survival than IRGPI-high patients.

**Conclusion:** Our findings indicated the potential value of the IRGPI risk model for predicting the prognosis of EC patients.

## Introduction

Esophageal cancer is a type of tumor with a very high mortality rate worldwide, with an increasing incidence rate in Western countries over the past few decades (Chen et al., 2021). EC patients have poor prognosis, with a 5-year survival rate lower than 15% (Jackie Oh et al., 2016), although clinical treatments have advanced rapidly (Kakeji et al., 2021). Chemoradiation is an optional treatment for resectable esophageal cancer to preserve the esophagus for patients who cannot tolerate surgery. Moreover, the combination of chemoradiotherapy and salvage surgery could extend the survival of patients (Kakeji et al., 2021). Esophageal carcinoma (EC) consists of two subtypes: esophageal adenocarcinoma (EAC) and esophageal squamous cell carcinoma (ESCC) (The Cancer Genome Atlas Research Network, 2017). In 2020, four clinical trials, CheckMate 649, ATTRACTION-4, KEYNOTE-590, and CheckMate 577, verified anti-PD-1 therapy as a first-line treatment for ESCC patients (Smyth et al., 2021). According to these latest results, esophageal adenocarcinoma cancer (EAC) may not be as sensitive to anti-PD-1 therapy as esophageal squamous cell carcinoma (Kelly, 2019). Immune infiltrating cells have been shown to be important to the response to immunotherapy. Previous studies have established IRG-based prognostic models for non-squamous non–small-cell lung cancer (Sun et al., 2020), ovarian cancer (Sun et al., 2020), breast cancer (Shen et al., 2019), colorectal cancer (Wang et al., 2020), osteosarcoma (Xiao et al., 2020), and bladder cancer (Li et al., 2021a). In this study, we established an IRGPI prognostic model and validated its role in different molecular features and prognoses in EC.

## Materials and methods

### Data source

RNA-seq data of 171 EC samples, including 160 cancer samples and 11 paracancer samples, and the matched clinical information were downloaded from The Cancer Genome Atlas (TCGA) database (https://portal.gdc.cancer.gov/). The GEO cohort (GSE53625) included 358 EC samples. The RNA-seq data and clinical information were downloaded from the Gene Expression Omnibus (GEO) database (https://www.ncbi.nlm.nih.gov/geo/).

The list of immune-related genes was downloaded from the ImmPort (https://www.immport.org/home) and InnateDB (https://www.innatedb.ca/) databases.

The regulatory relationships between mRNAs, transcription factors (TFs), and miRNAs were downloaded from the vBioPortal database (http://www.cbioportal.org/). The immune scores were computed using TIDE tools (http://tide.dfci.harvard.edu/).

### Differential expression analysis

Differentially expressed genes (DEGs) in cancer tissues compared to normal tissues were identified by the R package *limma*, with a false discovery rate < 0.05 and log2fold change >1.

### Enrichment analysis of immune-related genes

In functional enrichment analysis, the gene is selected between differentially expressed genes and immune-related genes. Gene Ontology (GO) and Kyoto Encyclopedia of Genomes (KEGG) enrichment analyses are run using the “clusterprofile” R package.

### Identification of immune-related hub genes

WGCNA was performed to identify hub genes that were significantly associated with EC (12). The simulation matrix was constructed by calculating Pearson’s correlation coefficients between two genes using RNA-seq data. Next, the similarity matrix was transformed into an adjacency matrix with a signed network type, and soft threshold *β* was set to 3 and then into a topology matrix, where topological overlap measure (TOM) was used to describe the degree of association between genes. The genes were clustered at a 1-Tom distance, and the dynamic pruning tree pair module was established for identification. Finally, the genes of the top 25% variance were filtered for further analysis in five modules (Chen et al., 2019). We chose two modules with *p* values lower than 0.05 to construct the network, and the genes in the network were hub genes. The *maxstat* R package was used to obtain the optimal cutoff value for each central gene to achieve overall survival (OS), and we obtained 21 genes that were significantly survival-associated, immune-related hub genes and thus selected for further analysis (*p* < 0.05, log-rank test).

### Establishment of the IRGPI model

The IRGPI model was established based on multivariate Cox regression analysis. Eight genes associated with overall survival were obtained from 21 immune-related hub genes. By summing the expression levels of the eight genes weighted by their Cox regression coefficients, we obtained an IRGPI model by which a risk score could be computed for each patient. Based on the IRGPI model, patients were stratified into high- and low-risk subgroups by median risk score. Through the calculation of multivariate Cox regression analysis, we can get the model formula of both the training group and the test group. By sorting out the clinical data set of the GES53625 data set, we can get two key pieces of information: survival time and survival state. Next, we extract the expression of model genes and obtain the risk score of the test group. Then, we can divide the test group into high- and low-risk groups according to the median value of the risk score. Kaplan–Meier (KM) survival analysis was used to evaluate the prognostic capacity of the IRGPI in TCGA and GEO cohorts.

### The molecular immune characteristics and ICI treatment of different IRGPI subgroups were comprehensively analyzed

To identify the immune microenvironment of 171 samples of EC, we used

CIBERSORT (https://cibersort.stanford.edu/) to estimate the relative proportion of 21 types of immune cells. Next, further analysis was conducted for the relative proportions of 21 immune cells and clinicopathological factors between the two IRGPI subgroups. We performed ssGSEA for genetic traits and compared scores between two IRGPI subgroups to further define their immune-related functions.

### Survival and Cox regression analysis

Kaplan–Meier survival analysis was performed by using the R packages “survival” and “surviviner”. Univariate and multivariate Cox regression analyses were conducted in order to identify the independent risk factors for prognosis. The forest maps were constructed by the R package “forestplot”, which showed the *p*-value and HR (95% CI) of each immune-related gene.

### Statistical analysis

Significance was considered as follows: *p*-value < 0.05 was considered statistically significant and highlighted by an asterisk in the figures, while *p* values < 0.01 were highlighted using two asterisks, and *p* values < 0.001 were highlighted using three asterisks in the figures.

## Results and discussion

### Identification of immune-related differentially expressed genes

**FIGURE 1:**
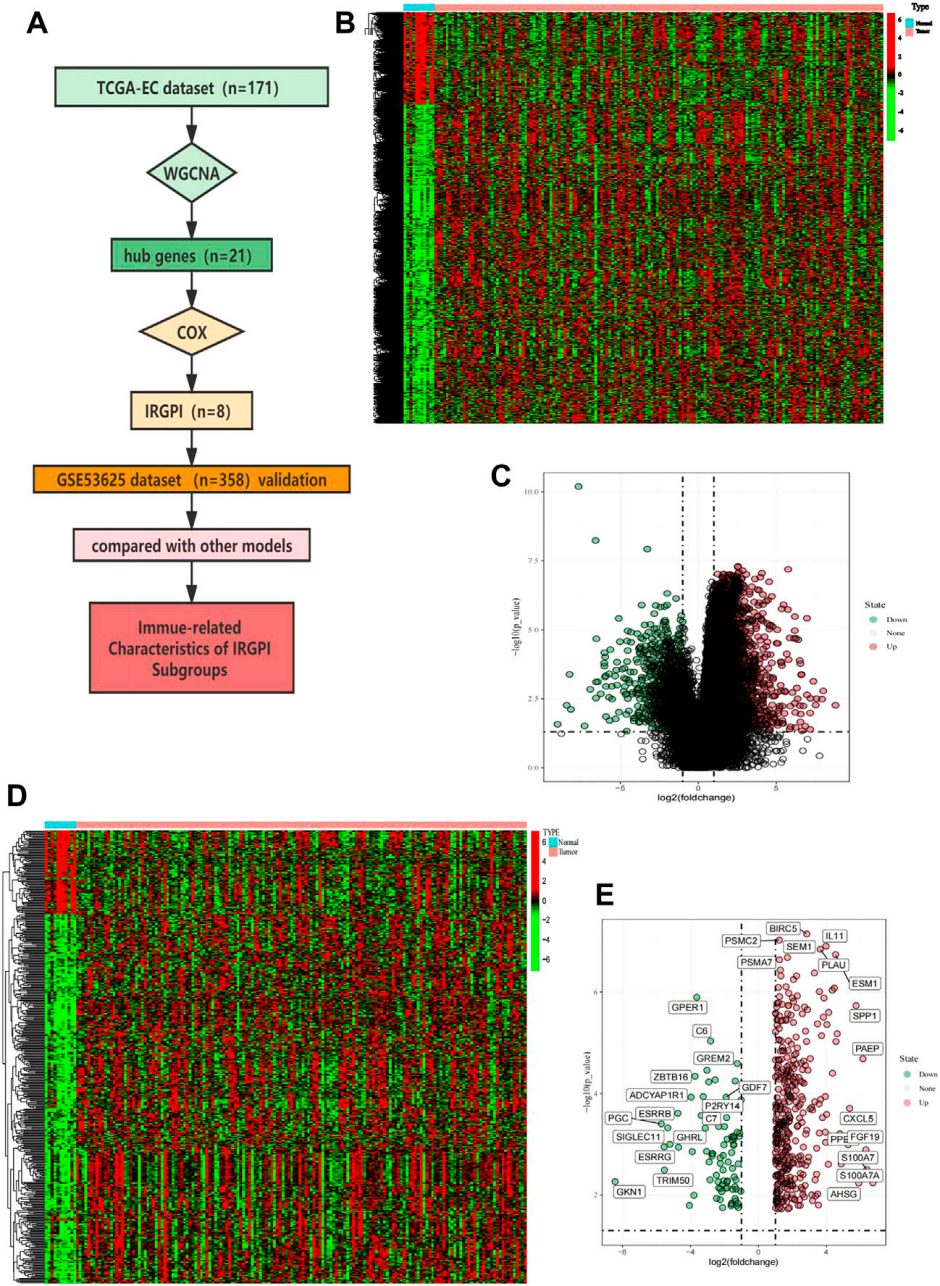
Overall analysis workflow and selected IRGs. **(A)** Schematic flowchart of the workflow performed to build and validate the EC prognostic model. **(B)** Heatmap of DEGs; red plots: cancer sample; green plots: normal sample; black plots: normally expressed mRNAs. **(C)** Volcano plot of DEGs; red plots: upregulation. Green plots: downregulation. **(D)** Heatmap of immune-related IRGs; red plots: cancer sample; green plots: normal sample; black plots: normally expressed mRNAs. **(E)** Volcano plot of IRGs; red plots: upregulation. Green plots: downregulation.

In the TCGA cohort that included 160 cancer samples and 11 normal samples (Figure 1B), we obtained 4,534 differentially expressed genes, including 3,519 upregulated genes and 1,015 downregulated genes, in the cancer samples compared to normal samples (Figure 1C). Taking the intersection of the immune genes collected from InnateDB and ImmPort, 446 IRGs were obtained (Figure 1D), of which 363 genes were upregulated and 83 genes were downregulated (Figure 1E).

### GO and KEGG enrichment analysis of IRGs

**FIGURE 2:**
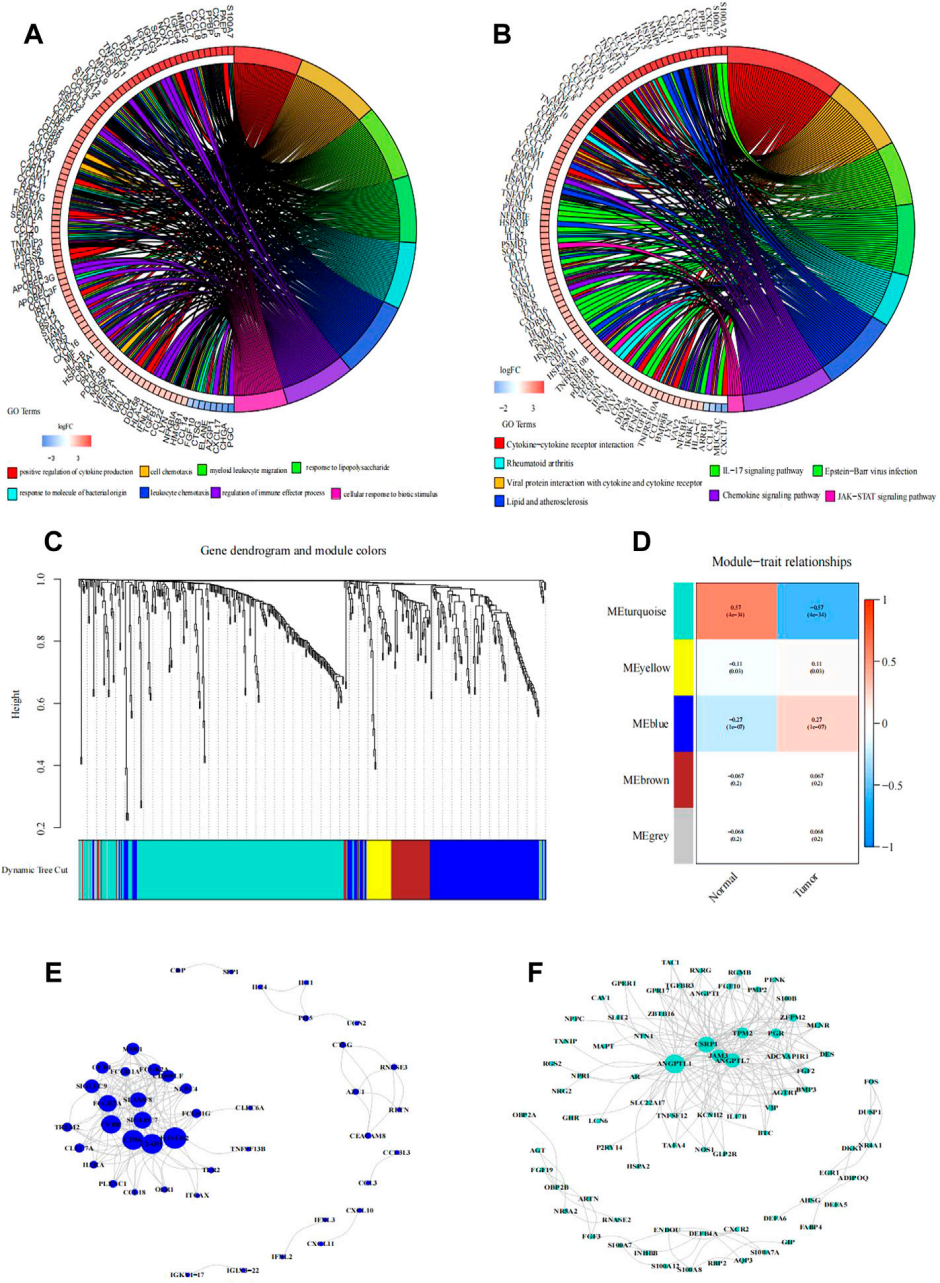
Functional enrichment analysis of differentially expressed IRGs. **(A,B)** Right shows significantly enriched GO or KEGG terms. Each bar on the left represents a gene, and the depth of the color represents the logFC value of the gene. The intermediate line represents the connections between genes and GO or KEGG terms. Identification of immune-related hub genes. **(C–F)** Gene dendrogram and module colors. **(D)** Module-trait relationships. WGCNA of immune-related DEGs with the soft threshold β = 3. **(E)** Network of the genes in the blue module (Weight of edge > 0.2). **(F)** Network of the genes in the turquoise module (Weight of edge > 0.2). The size of the circle indicates the number of genes in the enrichment pathway, the color of the circle indicates the approximation between different pathways, and the link indicates the genes in the enrichment pathway.

**FIGURE 3:**
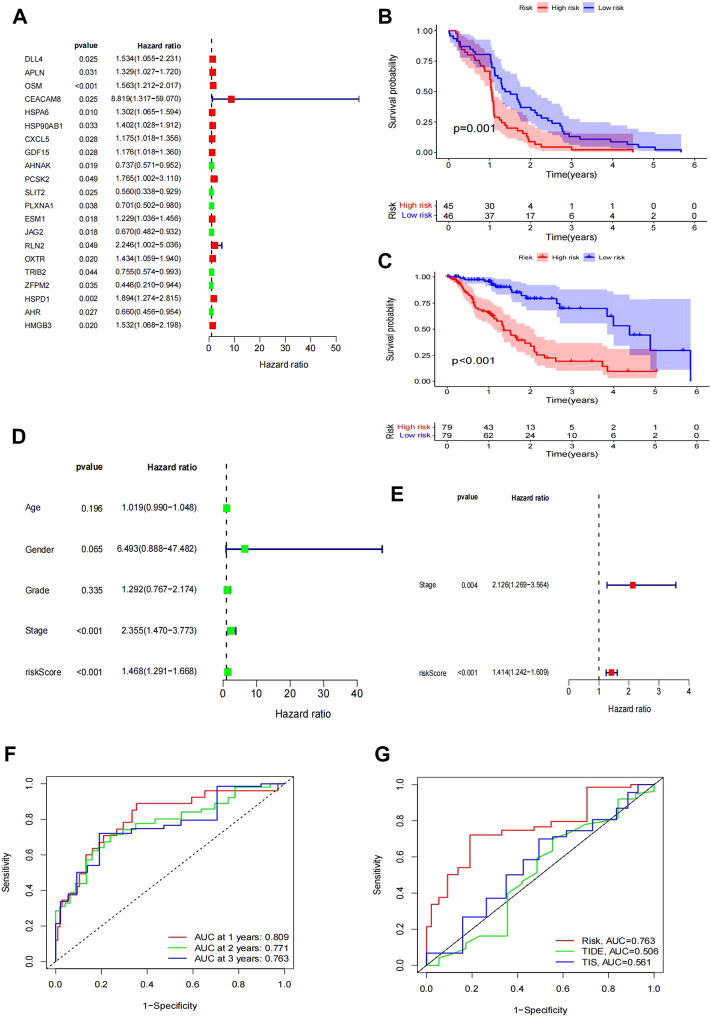
Construction of the IRG signature as a prognostic model. **(A)** Forest plot of hazard ratios showing the prognostic values of genes, in which the unadjusted hazard ratios, as well as the corresponding 95% confidence intervals, are displayed. **(B–C)** Survival plot of patient prognosis. **(B)** Survival analysis between the high-risk group and low-risk group of the TCGA test group. **(C)** Survival analysis between the high-risk group and low-risk group of the GEO training group. Forest plot of univariate and multivariate Cox regression analyses. **(D–E)** Uni-forest of the clinicopathological parameters: age, sex, grade, stage, and risk score of 171 EC patients. **(E)** Multi-forest of the clinicopathological parameter stage and risk score of the eight-gene module. **(F–G)** Comparison of the modules we established. **(F)** ROC curve lines of the patient at 1 year (*p* = 0.809), 2 years (*p* = 0.771), and 3 years (*p* = 0.763). **(G)** Comparison of the curve under the risk AUC (*p* = 0.763), TIDE AUC (*p* = 0.506), and TIS AUC (*p* = 0.561) samples.

The results of GO functional enrichment analysis are shown in Figures 2A and 3B. The GO analysis results illustrated that these IRGs were mostly involved in the positive regulation of cytokine production in biological processes (BP), the external side of the plasma membrane in cellular component (CC), and receptor–ligand activity in molecular function (MF) (Supplementary Figures S1A, B). The upregulated IRGs were enriched in the regulation of cytokine production, cell chemotaxis, myeloid leukocyte migration, and response to lipopolysaccharide, while the downregulated IRGs were enriched in the response to molecules of bacterial origin, leukocyte chemotaxis, regulation of immune effector processes, and cellular response to the biotic stimulus (Figure 2A).

The KEGG analysis results showed that the majority of the interactions were cytokine–cytokine receptor interactions (Supplementary Figures S1C, D). The positively correlated pathways included cytokine–cytokine receptor interactions, viral protein interactions with cytokines and cytokine receptors, the IL-17 signaling pathway, and Epstein−Barr virus infection (Figure 2B). The negatively correlated pathways included rheumatoid arthritis, lipids, atherosclerosis, the chemokine signaling pathway, and the JAK-STAT signaling pathway.

### Establishment of the IRGPI risk model

Based on the WGCNA results of 446 IRGs, we obtained 21 immune-related hub genes. As shown in Figures 2C and D, according to the correlation coefficient between each gene module and ESCC, we chose the turquoise and blue modules (correlation coefficient with EC > 0.6) for further analysis. The optimal soft-thresholding power was set to 3 based on the scale-free network (Figures 2D, E). After univariate Cox regression, 349 genes in the blue and turquoise modules were filtered out. Next, 21 genes significantly related to patient prognosis were selected by K-M analysis (Supplementary Figure S3, *p* < 0.05, log-rank test). Furthermore, multivariate Cox regression analysis of the 21 immune-related genes yielded eight genes that were finally used to build the risk model (Figure 3A). Formally, we computed the risk score as the weighted sum of their expression levels. Its formula is “OSM*0.50036 + CEACAM8*2.12798 + HSPA6*0.20461 + HSP90AB1*0.38072 + PCSK2*0.61100 + PLXNA1*-0.50040 + TRIB2*-0.43663 + HMGB3*0.47295”, in which the coefficients were derived from the Cox proportional hazard model.

### Validation of the IRGPI risk model

According to the median risk score as a cutoff value, the TCGA samples were divided into high- and low-risk subgroups. Survival analysis between the two subgroups showed that the low-risk group had a remarkably better prognosis than the high-risk group (Figure 3B). In the GEO GSE53625 cohort, we confirmed the prognostic value of the IRGPI risk model (Figure 3C).

Moreover, we compared the IRGPI risk scores and TIDE scores (http://tide.dfci.harvard.edu/) using the timeROC R package. The ROC curves for 1, 2, and 3 years are shown in Figure 3G. The ROC–AUC for 1-year OS prediction had the best performance**.** Additionally, our IRGPI model obtained better predictive power than the TIDE and TIS scores (Figure 3F).

For further study, we tested whether the IRGPI could be used as an independent biomarker with clinical significance. Therefore, we analyzed the clinicopathological parameters that influenced the survival outcome of EC patients, including age, sex, grade, and stage. The univariate Cox regression results showed that the HR of the IRGPI risk score was 1.468 (Figure 3E). However, other clinical variables, including age, sex, and grade, were not significant for OS. Moreover, the results of multivariate Cox regression verified that the HR of the IRGPI risk score was 1.1414, apart from the stage (HR = 2.126) (Figure 3E). These results showed that the IRGPI risk score was an independent risk factor for EC patients.

**FIGURE 4:**
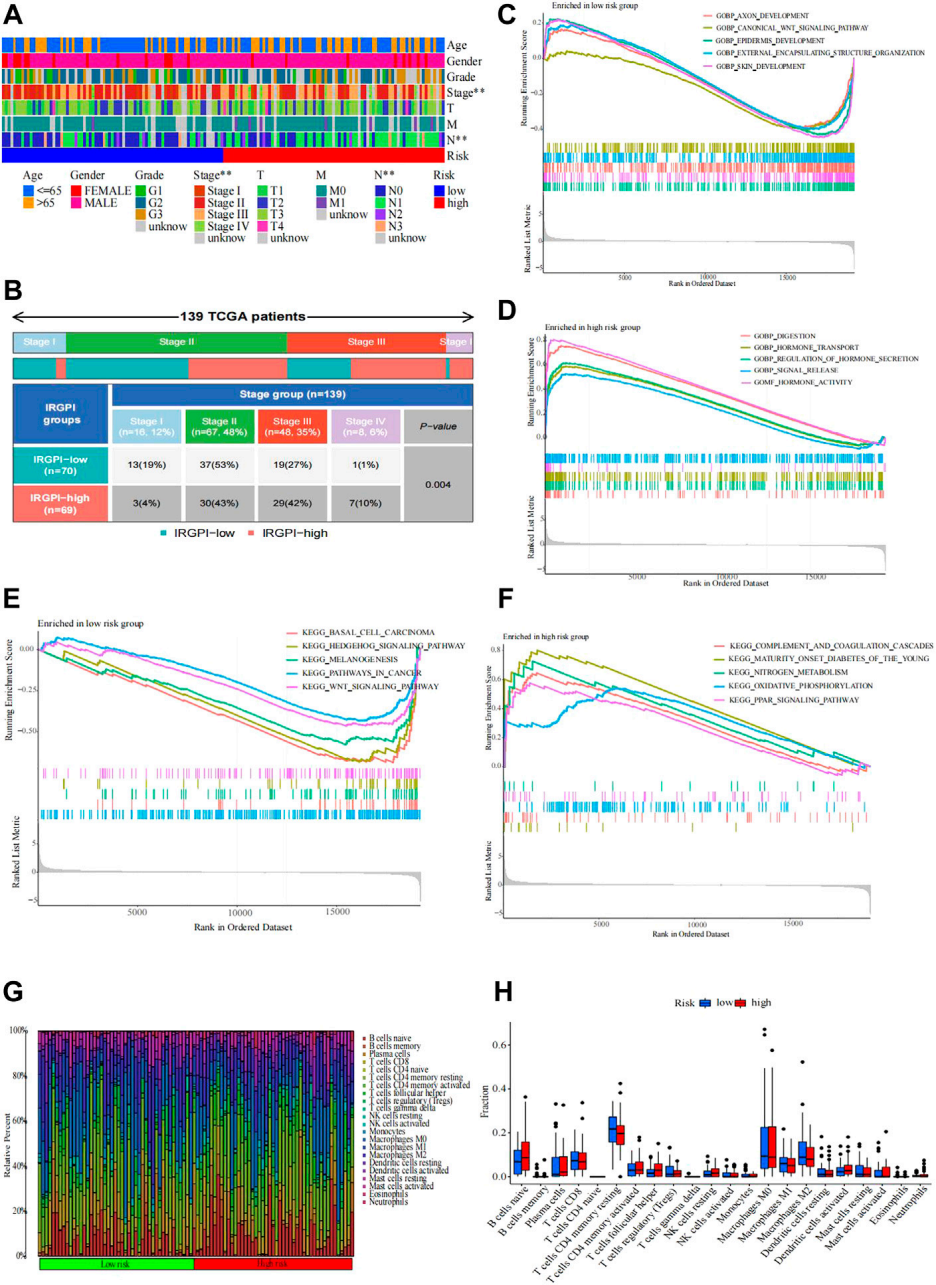
Distribution causes that affect patient prognosis. **(A,B)** Heatmap and table of the EC OS prognostic (age, sex, grade, stage, T, M, and N) between the IRGPI subgroups. **(B)** Heatmap and table of the stage between the IRGPI subgroups, and the distribution was compared through the χ2 test (*n* = 139, *p* = 0.004 < 0.05). Molecular characteristics of different IRGPI subgroups in GO and KEGG enrichment analyses. **(C–F)** GO enrichment analysis of gene sets enriched in the IRGPI-low and IRGPI-high subgroups (*p* < 0.05, FDR <0.25). **(F)** KEGG enrichment analysis of gene sets enriched in IRGPI-low and IRGPI-high subgroups (*p* < 0.05, FDR <0.25). Pattern of the TME and characteristics of different IRGPI subgroups in esophageal cancer. **(G–H)** Proportion of TME cells in different IRGPI subgroups. **(H)** Immune cell IRGPI subgroups. Scatter points represent the immune scores of the two subgroups. Thick lines represent the median. The bottom and top of the box are the 25th and 75th percentiles (interquartile range), respectively. Significant differences between the two subgroups were assessed using the Wilcoxon test (ns, not significant; *, *p* < 0.05; **, *p* < 0.01; ***, *p* < 0.001; ****, *p* < 0.0001).

We used the Wilcoxon test to test whether the clinical stage was still a prognostic marker within the two IRGPI subgroups. We found that clinical stage was a significant factor in the high- and low-IRGPI subgroups (Figure 4A). In addition, we checked the clinical stage by the RColorBrewer R package and found that stage II accounted for the largest proportion between the subgroups of IRGPI (*n* = 67.48%) and stage I accounted for the smallest proportion between the subgroups of IRGPI (*n* = 8.6%) (Figure 4B, *p* = 0.004, χ2 test).

### Immune microenvironment of IRGPI subgroups

The gene sets enriched in different IRGPI subgroups were detected by GSEA and analyzed by the “clusterprofile” R package (*p* < 0.05 and FDR <0.25). By GO enrichment analysis, we found that the gene sets of the low-IRGPI samples were enriched in axon development, canonical WNT signaling pathway, epidermis development, external encapsulating structure organization, and skin development, while the gene sets of the high-IRGPI samples were enriched in indigestion, hormone transport, regulation of hormone secretion, signal release, and hormone activity.

Next, KEGG enrichment analysis showed that the gene sets of the low-IRGPI sample were enriched in basal cell carcinoma, the Hedgehog signaling pathway, melanogenesis, pathways in cancer, and the WNT signaling pathway. The gene set of the high-IRGPI samples was enriched in complement and coagulation cascades, maturity-onset diabetes in young people, nitrogen metabolism, oxidative phosphorylation, and the PPAR signaling pathway (Figures 4C–F).

**FIGURE 5:**
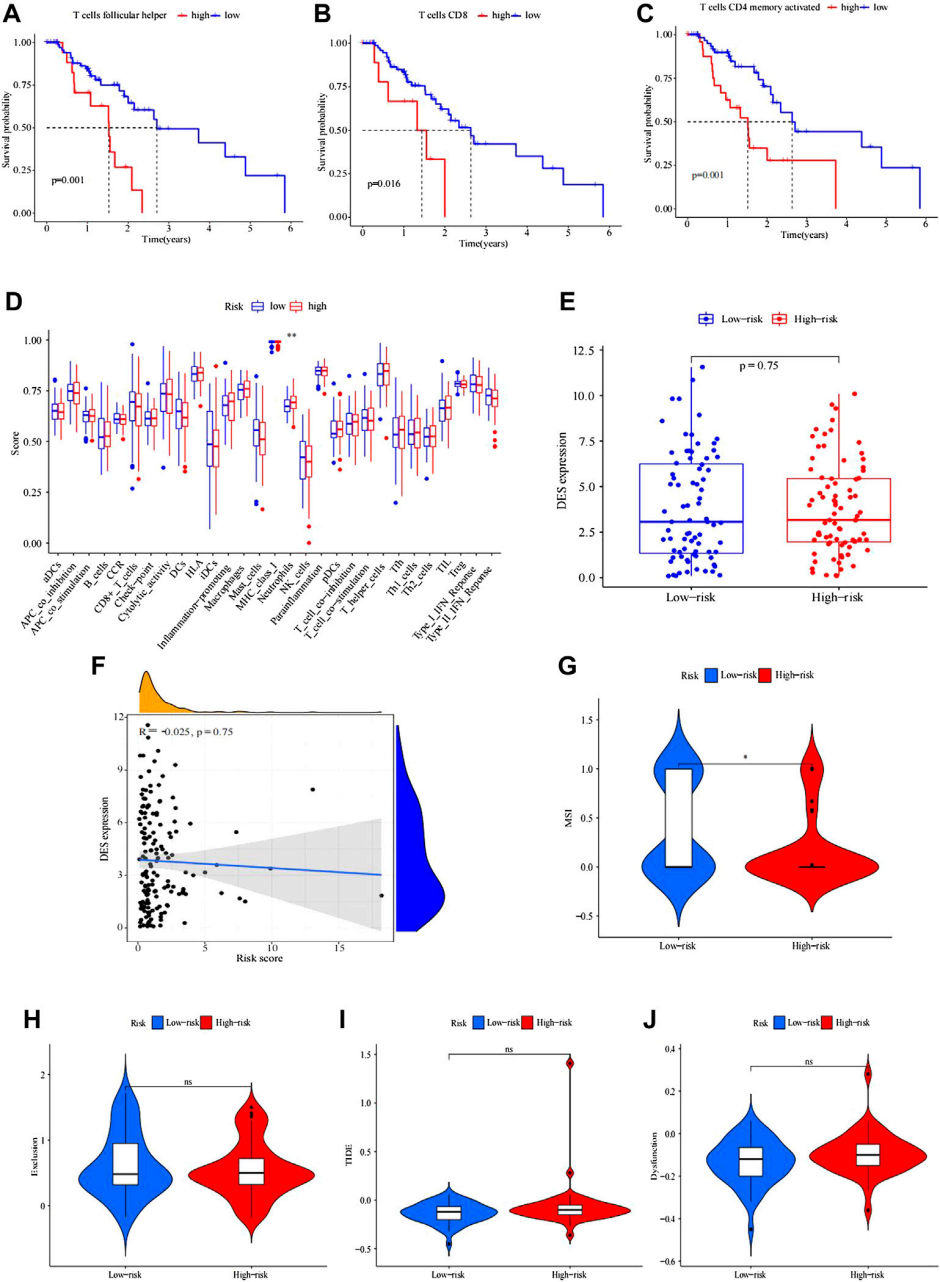
Kaplan–Meier survival analysis of immune-related cells in IRGPI subgroups. **(A–C)** Differences in follicular helper T cells between IRGPI subgroups. **(B)** CD8 T cells were different between IRGPI subgroups. **(C)** CD4 memory-activated T cells differed between the IRGPI subgroups (*p* < 0.05). **(D)** Molecular- and immune-related functions of different IRGPI subgroups. The molecular- and immune-related gene sets of the IRGPI were analyzed by single-sample gene set enrichment analysis (ssGSEA), and the differences between different IRGPI subgroups were compared. Scatter points represent the ssGSEA scores of the two subgroups. Thick lines indicate median values. The bottom and top of the box are the 25th and 75th percentiles (quartile range), respectively. Significant differences between the two subgroups were tested by the Wilcoxon test (NS: not significant, * * *p < 0.05, *p* < 0.01, ***p < 0.001). Analysis of mutation load difference in the tumor. **(E–F)** Boxplot of differences in tumor mutation load between high and low IRGPI. **(F)** Correlation test analysis of patient risk score and tumor mutation burden. (*p* = 0.023, R = 0.18) Immune escape and immunotherapy of TIDE, MSI, exclusion, and dysfunction score in different IRGPI subgroups. **(G–J)** Violin plot of exclusion between the IRGPI subgroups. **(H)** Violin plot of dysfunction between the IRGPI subgroups. **(I)** Violin plot of MSI between the IRGPI subgroups. **(J)** Violin plot of TIDE scores between the IRGPI subgroups. The scores between the two IRGPI subgroups were compared through the Wilcoxon test (ns, not significant; *, *p* < 0.05; ***, *p* < 0.001).

To analyze the composition of immune cells in different IRGPI subgroups, we visualized the immune microenvironment of the two subgroups (Figure 4G). The results showed that the proportions of infiltrating immune cells between the IRGPI subgroups were not different (Figure 4H). Moreover, immune cells associated with EC prognosis of the IRGPI subgroups were assessed by Kaplan–Meier (KM) survival curves with log-rank tests. We found that T follicular helper cells (*p* = 0.001), CD8 T cells (*p* = 0.016), and activated memory CD4 T cells (*p* = 0.001) were different between the low-IRGPI and high-IRGPI groups (Figures 5A–C).

ssGSEA was applied to analyze immune cell infiltration in tumors in the TCGA cohort by using immune-related genes (Figure 5D). We used the Wilcoxon rank test to distinguish the difference in immune cell infiltration between IRGPI subgroups. We found that the immune-related functions of DCs, macrophages, neutrophils, parainflammation, and T helper cells were different between the high- and low-risk groups, and these cells were more abundant in the high-IRGPI subgroups. By using K-M survival analysis, B cells, checkpoints, macrophages, mast cells, neutrophils, T-cell coinhibitory molecules, Th2 cells, TILs, and the type II IFN response were obviously associated with prognosis in the IRGPI in the high- and low-risk subgroups (Figures 5A–C).

There was no difference in tumor mutation load between the high- and low-risk groups of IRGPI (*p* = 0.1). The correlation tests showed that there was a positive correlation between risk score and patient tumor mutation load (*p* = 0.023, *R* = 0.18).

The potential immunotherapy benefit was evaluated by using the TIDE R package.

We explored the potential clinical efficacy in the IRGPI high- and low-risk groups (Figures 5E, F). In general, a lower tide prediction score indicated a lower possibility of immune escape and a higher benefit from ICI treatment. Higher TIDE prediction scores were associated with poorer outcomes. In our results, TIDE exclusion and dysfunction scores were not significantly different between the IRGPI high- and low-risk groups (Figures 5G–J), but the MSI score of the low-risk group was higher, indicating that the low-risk group was more sensitive to immunotherapy (Figure 5G).

The patient conditions would be improved.

## Discussion

Esophageal cancer, as the seventh most common cancer, has poor prognosis and higher mortality. At present, in the area of immune-related therapy, EC patients have three research orientations: active immunization, passive immunization, and inhibition of immune checkpoints (ICIs). The immune checkpoint inhibitor (ICI) in EC has been approved by the United States Food and Drug Administration. However, the efficacy of ICIs in low PD-L1-expressing tumors remains unclear, and by using K-M subtraction, in low PD-L1-expressing GEAC tumors, there was a lack of benefit from the addition of ICI to chemotherapy (Zhao et al., 2022). The safety and efficacy of anti-PD-1 antibodies, including pembrolizumab and nivolumab, for esophageal cancer and the anti-CTLA-4 antibodies (ipilimumab) and anti-PD-1 antibodies (nivolumab) in advanced CTLA-4 in late esophageal cancer have been significantly demonstrated in recent clinical trials (Huang and Fu, 2019). During this period, some publications have presented that in the groups of EACs, T-cell-rich inflammation has an outstanding prognostic correlation (Schoemmel et al., 2021). In the area of immune-related therapy for colorectal cancer, the immune score is a stronger predictor of patient survival than microsatellite instability (Mlecnik et al., 2016). The clinical prognosis of esophageal cancer is relatively unfavorable due to lack of efficient early screening and diagnosis and limited therapeutic options. In addition, due to limited efficacy and drug resistance of immunotherapy, radiotherapy, and chemotherapy, establishing an immune-related gene prognostic index is a direction worth navigating. The prognostic model of EC we established has been continuously updated for the eight genes we selected by using WGCNA. These genes, namely, OSM, CEACAM8, HSPA6, HSP90AB1, PCSK2, PLXNA1, TRIB2, and HMGB3 have a significant effect on patient OS. Our study takes into account the comparison of the ROC line of the IRGPI. The results of comparative ROC lines show that the model we constructed has a high degree of accuracy, and we also used the GSE53625 (*n* = 358) database to verify the accuracy of the model. Moreover, we also conclude that the IRGPI could be a prognostic immune-related biomarker for esophageal cancer since the model showed better survival in IRGPI-high EC patients and worse survival in IRGPI-low EC patients in both the TCGA and GEO cohorts.

Additionally, according to the clinically relevant heatmap of IRGPI subgroups by the ComplexHeatmap package of R, we learned that the patient’s clinical stage was different between the high- and low-risk groups and could be an important factor affecting EC patient OS.

For further study, we explored the molecular characteristics of different IRGPI subgroups through GSEA enrichment analysis. According to a previous study, the high serum Wnt signaling antagonist DickkopF-associated protein 1 is associated with impaired overall survival and recurrence in patients with esophageal cancer (Ramirez et al., 2021) and the biological process of significant overexpression of downregulated genes in epidermal development (Fu et al., 2015). According to recent studies, in EC skin development, COX-2 can promote the initiation of invasive tumor formation in tumor-prone dry/progenitor cells in mouse skin and the formation of esophageal SCC at the squamous junction (Moon et al., 2020). The treatment of esophageal cancer has a hormone level of E2 that can be used to treat reflux esophagitis, achalasia of the cardia, esophageal cancer, and other esophageal diseases (Kim et al., 2017). Exosome incubation and xenotransplantation experiments indicated that fMR1-AS1 exosomes might be secreted from ESCC CSCs, transferring the dry phenotype to recipient non-CSCs in the tumor microenvironment (Li et al., 2019a). In addition, we found a correlation between serum levels of FMR1-AS1 and overall survival (OS) in women with ESCC (Li et al., 2019a). Mir-135a inhibits the invasion and migration of esophageal cancer stem cells by targeting the Smo Hedgehog signaling pathway (Yang et al., 2021). Radiotherapy plays an important role in the treatment of esophageal cancer in general. In radiosensitivity studies of esophageal cancer, circRNA_100367 silencing inhibited the proliferation and migration of KYSE-150R cells and reduced the expression of β-catenin (an important molecule in the Wnt pathway) in KYSE-150R cells. In addition, circRNA_100367 binds to miR-217, which targets Wnt3. Low Wnt3 expression was associated with shorter survival time in ESCC patients, and Wnt3 knockdown inhibited the proliferation and migration of KYSE-150R cells (Liu et al., 2019). In the nitrogen metabolism enrichment analysis, nitrotyrosine is a product of nitrogen and is expressed in esophageal squamous cell carcinoma, suggesting that exogenous risk factors such as tobacco and alcohol are associated with the occurrence and progression of esophageal squamous cell carcinoma through NO (Kato et al., 2000). Recent advances have revealed a novel redox homeostasis signaling pathway that regulates the pathologic biology of ESCC and identified IFI6 as a potential drug target in ESCC. In summary, the LINC00184/PTEN/Akt axis mediates glycolysis and mitochondrial OXPHOS in EC cells. This study highlights potential intervention targets for the treatment of EC (Li et al., 2019b; Liu et al., 2020). Moreover, the PAR signaling pathway illustrates that PPAR gamma antagonists inhibited the invasion and cell adhesion of esophageal carcinoma cells, probably due to alteration of the FAK-MAPK pathway, which was unrelated to apoptosis. The results also suggest that PPARγ plays an important role in the invasion of cancer cells and may be a new target for the treatment of esophageal cancer (Takahashi et al., 2006). The underlying mechanism by which the IRGPI was enriched remains unclear and needs further study. Therefore, studying these identified signaling pathways may shed light on the carcinogenic mechanisms behind EC.

Microsatellite instability is a biomarker of PD-1 blockade. Tumor types can be classified according to the frequency of MSI, from colorectal cancer (20%) and endometrial cancer (22–33%) to cervical cancer (8%) and esophageal cancer (7%) to skin cancer and breast cancer (0–2%). At present, MSI can be used as one of many biomarkers to guide the treatment decisions of patients with esophageal and gastric adenocarcinoma, and MSI is the cause of neoplasms in colorectal, gastric, and endometrial cancers (Thibodeau et al., 1993; Liu et al., 1995; Liu et al., 1996; Wirtz et al., 1998; Halling et al., 1999; Goel et al., 2003; Boland and Goel, 2010). Microsatellite instability (MSI) due to mismatch repair defects is present in 4–20% of gastroesophageal cancers and is associated with favorable survival outcomes. This prognostic advantage may be related to immune surveillance; hence, the favorable response to immune checkpoint inhibition observed in tumors with high MSI (MSI-H) (Dudley et al., 2016; Dhakras et al., 2020; van Velzen et al., 2020).

In our study cohort, we found microsatellite unstable EACs in only 0.6%, which was published previously. More evidence shows that in order to fully understand the molecular composition of esophageal cancer, we should pay attention not only to tumor microscopy (TME) but also to tumor cells. Cell populations, such as suppressor cells and regulatory T cells from bone marrow, and immune checkpoints, such as programmed death 1, weaken antitumor immunity (Lin et al., 2016). IRGPI was made up of eight genes, OSM, CEACAM8, HSPA6, HSP90AB1, PCSK2, PLXNA1, TRIB2, and HMGB3. Among the emerging targets and biomarkers, the anticancer hormone (OSM) has attracted extensive attention in the past few years. OSM has diagnostic, prognostic, and therapeutic capabilities (Verstockt et al., 2019) and has been identified as an inhibitor of tumor cell growth in a variety of cancers, including melanoma, ovarian cancer, and glioblastoma cancer (Brown et al., 1987; Friedrich et al., 2001; Ohata et al., 2001; Tawara et al., 2018). Furthermore, CEACAM8 could be used to evaluate the relationship between clinicopathological features and prognosis of patients in the period study. For example, CEACAM8 is used as a risk signature for inflammation and T immune cell infiltration in colorectal cancer to predict distant metastasis and chemotherapy efficiency (Hu et al., 2019). CEACAM6 expression has also been implicated in bone metastasis of breast cancer, and the coexpression of CEACAM6 and 8 inhibits the proliferation and invasion of breast cancer cells (Iwabuchi et al., 2019). RNA sequencing revealed that heat shock 70-kDa protein 6 (HSPA6), a novel thymoquinone upregulation gene, inhibited the growth, migration, and invasion of triple-negative breast cancer cells (Shen et al., 2021). HSPA6 enhanced the inhibitory effect of garlic extract on the proliferation, migration, and invasion of bladder cancer EJ cells (Shin et al., 2017). Analysis of TCGA data showed that high HSP90AB1 expression was also associated with poor prognosis in breast cancer but with a better prognosis in rectal cancer patients (Uhlen et al., 2017). Hsp90ab1 is overexpressed and associated with poor prognosis, proliferation, and invasion of GC (Wang et al., 2019). Some data suggest that EXO-LNC RNA PCSK2-2:1 may play an important role in the progression of gastric cancer and can be used as a potential marker for diagnosis of gastric cancer. In addition, PCSK2 can also be used as an indicator to identify follicular variants of thyroid papillary carcinoma (Weber et al., 2005; Cai et al., 2019). The increased expression of PLXNA1 promoted the growth of prostate tumors and independently predicted the biochemical recurrence, metastasis, and poor survival of prostate tumors in a multi-institutional PCA patient cohort. Furthermore, PLXNA1 is also a promising therapeutic target for renal clear cell carcinoma (Ren et al., 2018; Li et al., 2021b). The characteristics of TRIB2 structure and signal transduction and its role in many cancer subtypes focus on the function of TRIB2 in the therapeutic resistance of melanoma, leukemia, and glioblastoma (Mayoral-Varo et al., 2021). In some studies, HMGB3 may be a useful prognostic indicator for patients with GC. In addition, the HMGB3/hTERT signaling axis can be used as a new target for radiation resistance in cervical cancer, which provides new insights into the antiradiation mechanism of cervical cancer and suggests that targeting the HMGB3/hTERT signaling axis may be beneficial to patients with cervical cancer (Fang et al., 2020; Li et al., 2020). Although there are many models associated with the prognosis of esophageal cancer, this is the first time that the WGCNA method has been used to establish an 8-gene model. This model does not require whole-genome sequencing for EC patients and is inexpensive and can predict patient prognosis at 1, 2, and 3 years, and the prediction effect is better when combined with patient stage. For the accuracy of our model, we used relevant datasets for verification and obtained good accuracy results. In addition, the methods used in this study may also apply to other types of malignancies.

At the same time, we recognize that there are local limitations to the model that we built. First, the experimental data were mainly derived from the TCGA database, and only the GEO database was used for validation, which was not verified in other databases or other clinical and pathological data. Second, we did not follow up on patient outcomes. Third, this study only proposed a preliminary prognostic model, the validity of the gene signature model needs to be further verified by clinical trials, and further functional studies are required to elucidate the underlying mechanisms of these eight genes.

## Conclusion

In our study, we established a novel eight immune-related gene model, which is a promising immune-related prognostic biomarker. Importantly, the IRGPI may help distinguish immune and molecular characteristics and predict patient outcomes. The IRGPI may be a potential prognostic indicator of immunotherapy, but further studies are needed to clarify this.

## Data Availability

The original contributions presented in the study are included in the article/Supplementary Material; further inquiries can be directed to the corresponding authors.

## Abbreviations

DEGs, differentially expressed genes; EC, esophageal cancer; EAC, esophageal adenocarcinoma; ESCC, esophageal squamous cell carcinoma; IRGs, immune-related genes; IRGPI, immune-related gene prognostic index; ICI, immune checkpoint inhibitor; OS, overall survival.
