# Supplementary figures and images for: Establish immune-related gene prognostic index for esophageal cancer
Source: Front Genet. 2022 Aug 9;13:956915. doi: 10.3389/fgene.2022.956915 (PMC9401516; doi:10.3389/fgene.2022.956915)

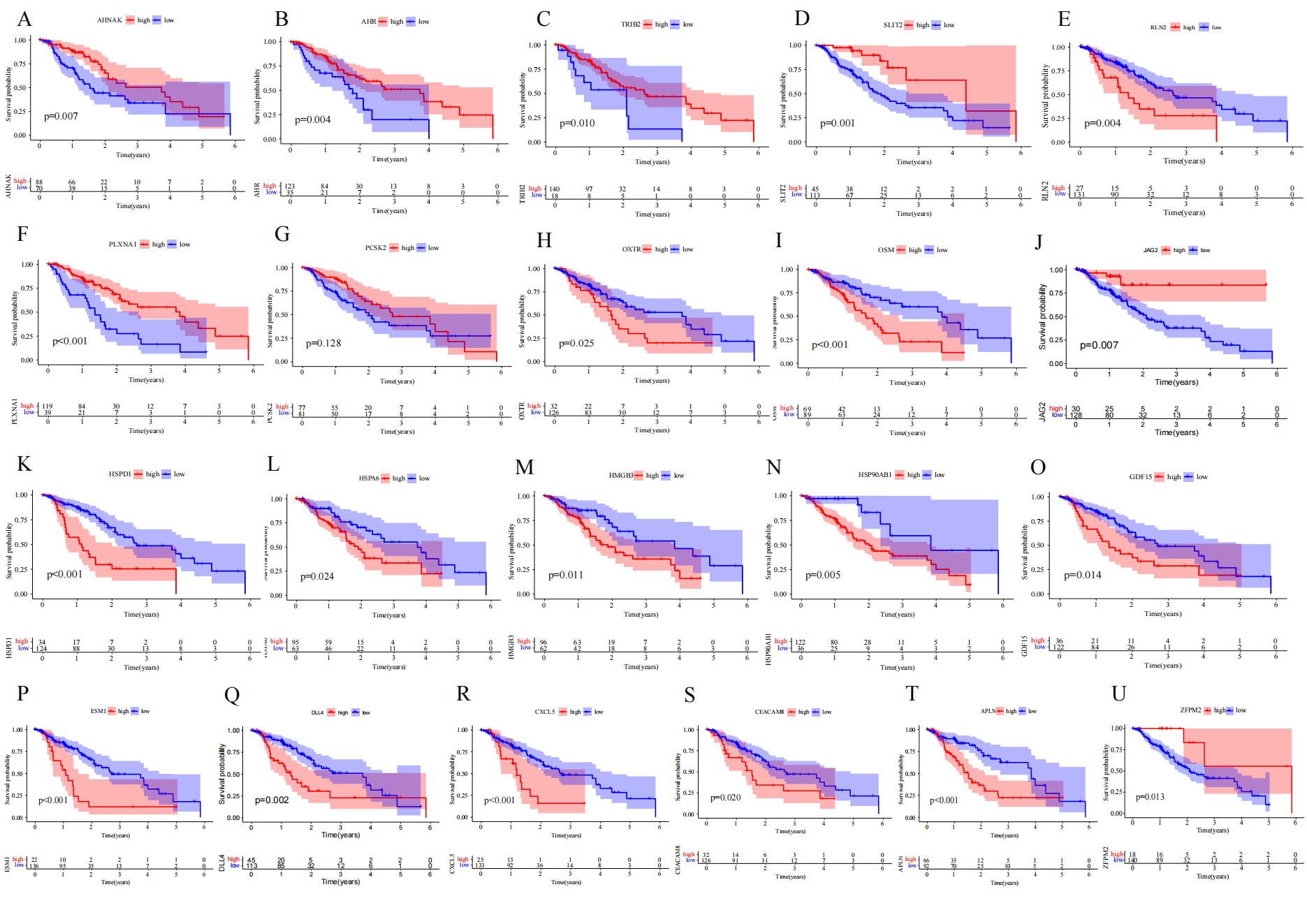

Supplement: Supplementary file 1 [file Image3.JPEG]

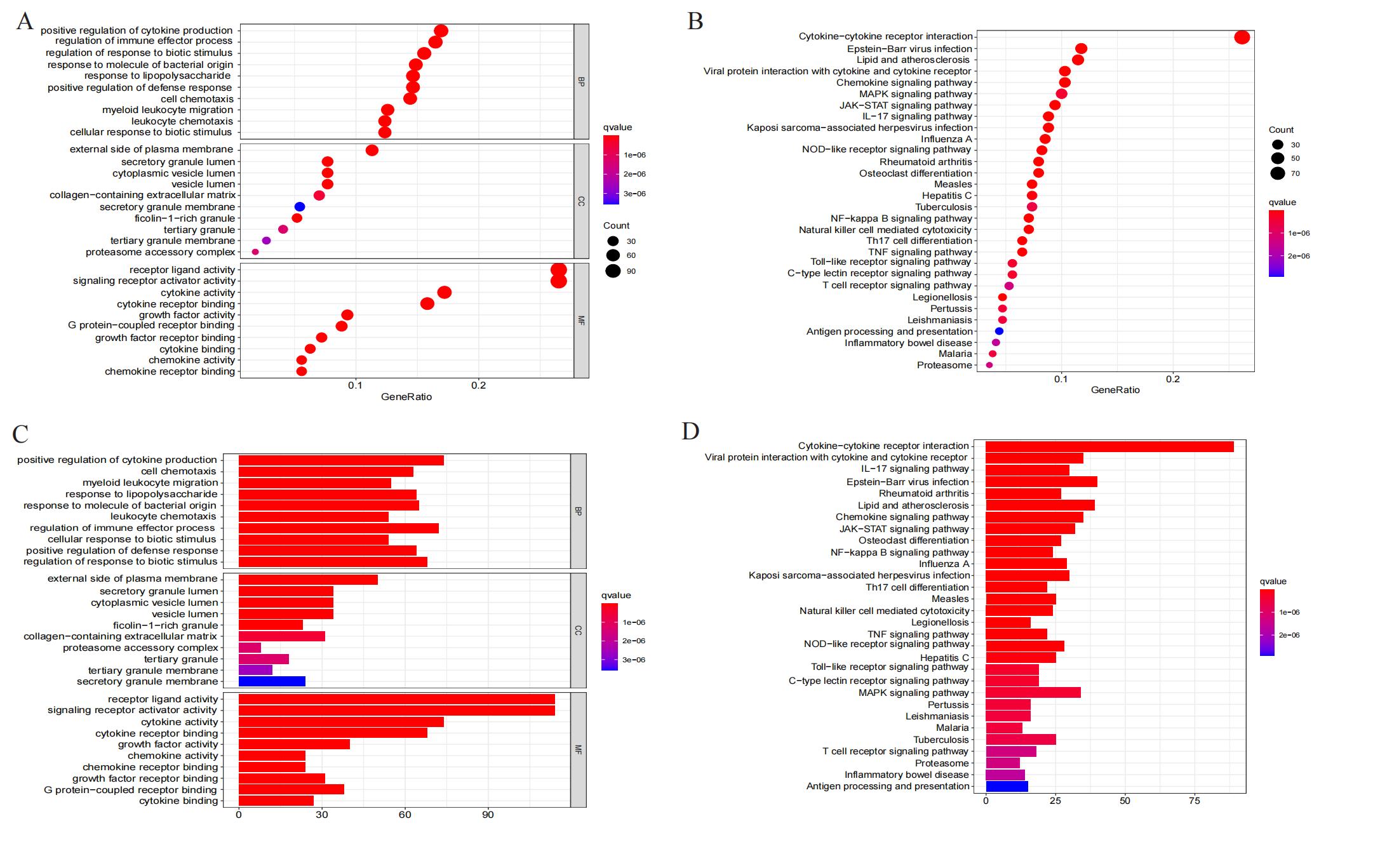

Supplement: Supplementary file 2 [file Image1.JPEG]

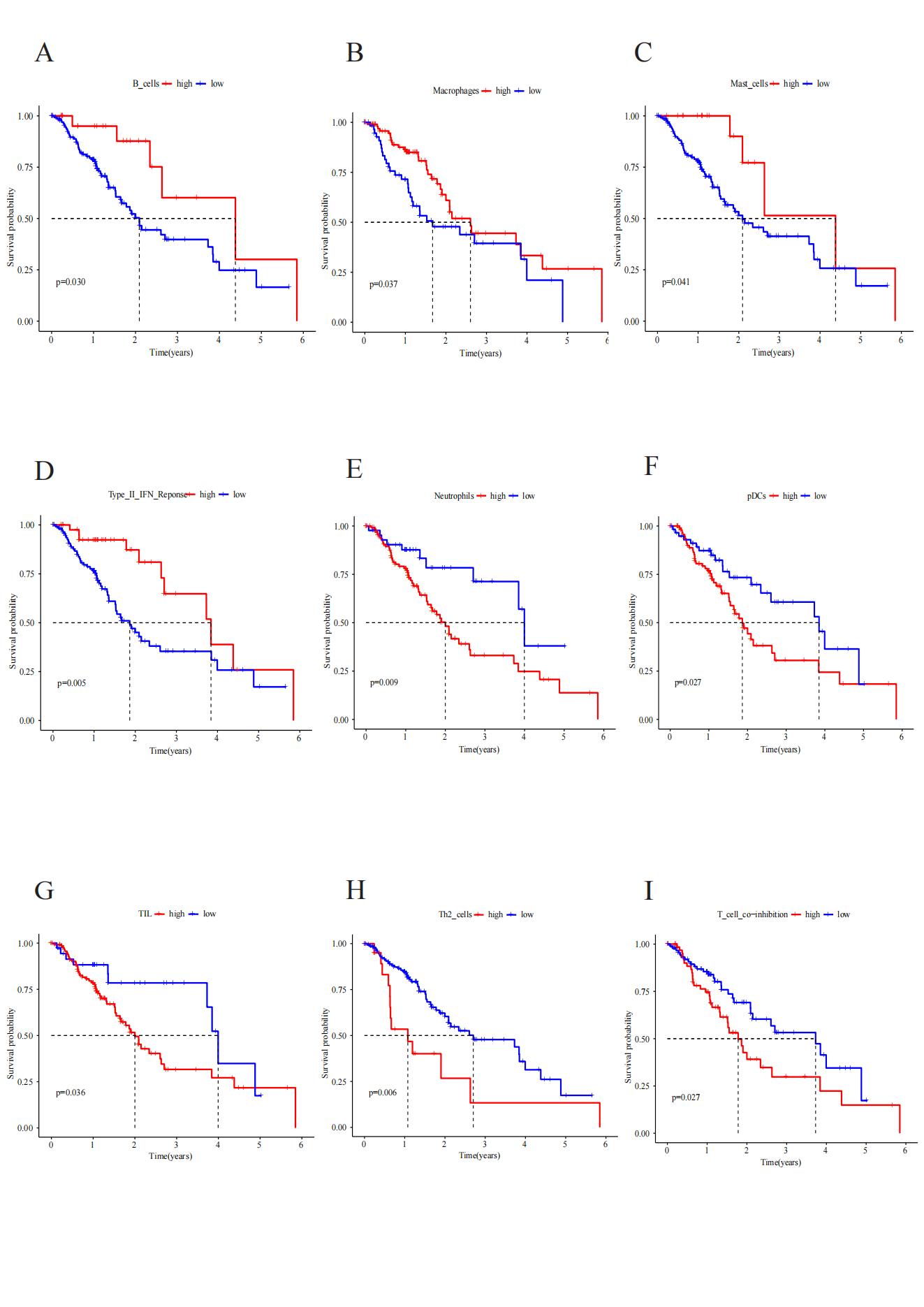

Supplement: Supplementary file 3 [file Image4.JPEG]

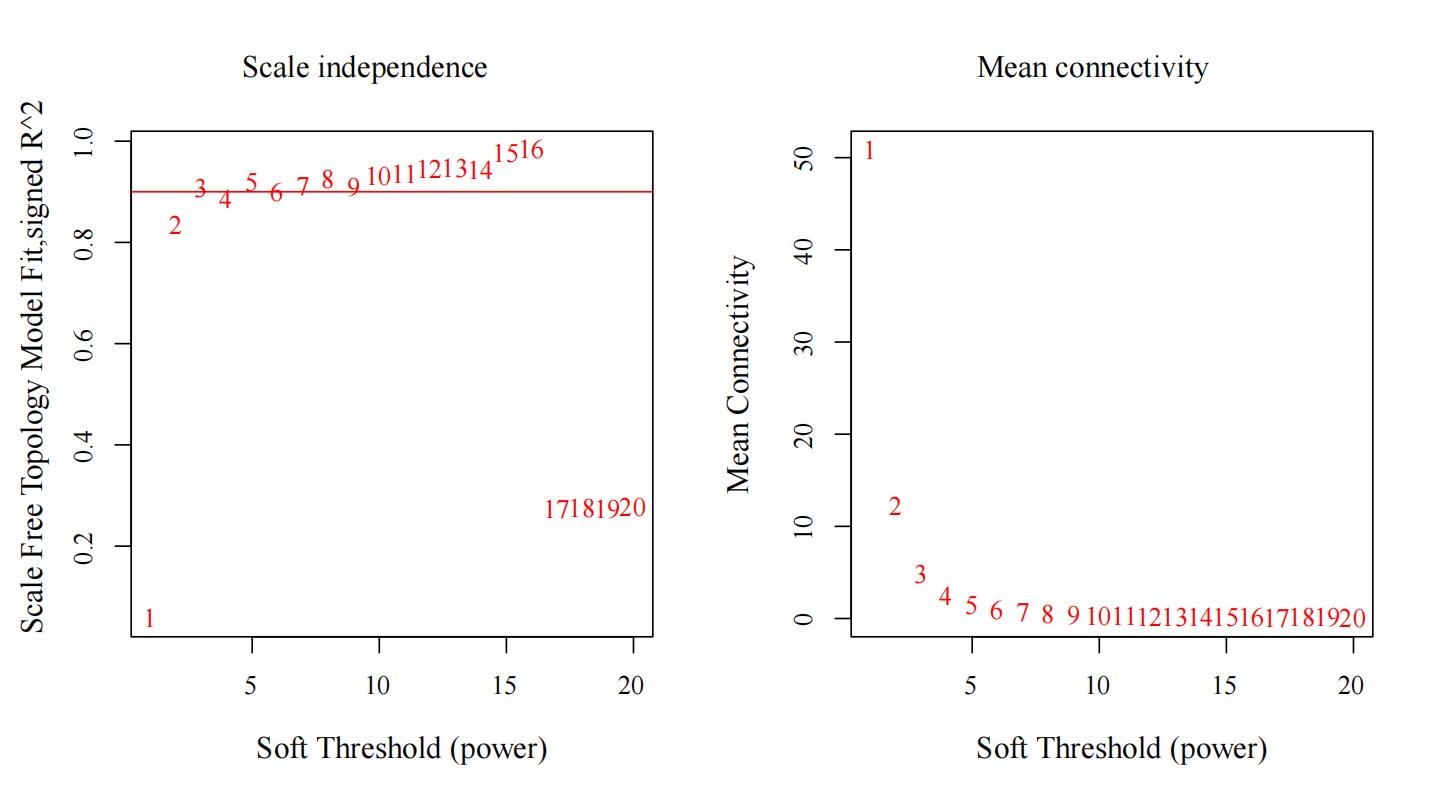

Supplement: Supplementary file 4 [file Image2.JPEG]
